# Loss of ENTV‐2 RNA and Point Mutation of *Env* Gene in ENA‐1 Cells During Successive Passages

**DOI:** 10.1155/av/4909539

**Published:** 2026-08-23

**Authors:** Mingxiao Li, Wenqing Guo, Ziyan Lin, Lingxu Li, Zhen Wang, Bingyan Wei, Guangjun Chang, Dawei Yao

**Affiliations:** ^1^ College of Veterinary Medicine, Nanjing Agricultural University, Nanjing 210095, China, njau.edu.cn

**Keywords:** *env* gene, enzootic nasal adenocarcinoma, enzootic nasal tumor virus 2, sequence analysis

## Abstract

**Purpose:**

This study aimed to monitor the presence of enzootic nasal tumor virus 2 (ENTV‐2) and the dynamic changes of the viral *env* gene during the successive passages of enzootic nasal adenocarcinoma (ENA) cells.

**Materials and Methods:**

ENA cells were passaged for 24 generations, and the supernatants and cell pellets were collected to extract RNA for monitoring viral presence. The ENTV‐2 *env* gene was amplified by PCR, cloned, and sequenced to analyze nucleotide mutations in the *env* gene sequence across different passages.

**Results:**

The results showed that ENTV‐2 RNA was consistently detected in ENA cells up to the 22nd passage, indicating sustained viral persistence within the cells, while its presence in the supernatant was unstable, suggesting possible regulation of viral release, although the underlying mechanism was not investigated. Further cloning and sequencing of the *env* gene from 11 passages revealed no deletions or insertions, but point mutations were observed, predominantly A ⟶ G substitutions, some of which led to amino acid changes. Notably, nonsense mutations W231 and W19 occurred in the 7th and 15th passages, respectively, resulting in premature truncation of the Env protein, while the Q168R substitution was consistently identified in the sequenced clones from all passages from Passage 5 through Passage 22. Structural predictions indicated that the Env protein maintained a conserved core structure with localized variations in non‐transmembrane regions. These observations are consistent with possible structural fine‐tuning, but functional validation is required.

**Conclusion:**

This study provides a descriptive account of sequence changes in the ENTV‐2 *env* gene during serial passaging of ENA cells. The findings are hypothesis‐generating and await experimental verification of their biological significance.

## 1. Introduction

Enzootic nasal tumor (ENT), also known as enzootic nasal adenocarcinoma (ENA), is a chronic, contagious, and highly fatal neoplastic disease caused by the enzootic nasal tumor virus (ENTV) [1]. The virus responsible for goat ENA is specifically termed the enzootic nasal tumor virus 2 of goats (ENTV‐2). ENTV‐2 is a betaretrovirus with a genome consisting of a single‐stranded, linear positive‐sense RNA dimer, approximately 7500 base pairs in length. Its genome structure includes noncoding long terminal repeats (LTRs) at both ends and a central coding region containing the *gag*, *pro*, *pol*, and *env* genes [2, 3]. Among these, the *env* gene plays a central role in viral pathogenesis. It encodes the viral envelope glycoprotein (Env), which comprises the surface (SU) and transmembrane (TM) subunits. This protein is crucial for mediating virus binding to host cell receptors, facilitating viral entry, enabling virion release, and contributing to both the initial infection process and subsequent oncogenesis [4]. Research has shown that the Env protein not only mediates viral recognition and attachment to host cells but also functions as a primary oncoprotein. It can directly induce cellular transformation and tumorigenesis by activating key cellular signaling pathways such as MEK–MAPK and PI3K–Akt [5, 6]. Therefore, an in‐depth study of the *env* gene is critical for elucidating the pathogenic mechanisms of ENTV‐2.

Building on this foundation, the present study involves the successive passaging of ENA cells harboring ENTV‐2. We systematically monitored the presence of viral RNA across passages, cloned and sequenced the viral *env* gene, and compared the sequence variations among different passages. We further analyzed the potential impact of genetic mutations on the structure and function of the viral protein. This work aims to provide a descriptive characterization of sequence stability and variation across passages.

## 2. Materials and Methods

### 2.1. Cell Culture Procedures

ENA‐1 cells (CCTCCC 2022232) were isolated from tumor tissues of ENA goat [7]. The cells at 3rd passage were cultured in complete RPMI‐1640 medium (11875, Solarbio, Beijing, China) supplemented with 10% fetal bovine serum (FB25015, Clark Bioscience, California, USA), 100 U/mL penicillin, 0.1 mg/mL streptomycin, and 0.2 mg/mL ceftiofur sodium. Cells were routinely cultured in an incubator at 37°C in 5% carbon dioxide. At 90% confluency, the cells were washed twice with 3 mL phosphate buffer (D‐PBS, D1040, Solarbio, Beijing, China) and were detached with 1 mL 0.25% trypsin (T1350, Solarbio, Beijing, China) for 5 min at 37°C and then gently resuspended in 5‐mL complete RPMI‐1640 medium. The cells were centrifuged at 1200 r/min for 5 min and resuspended in 1‐mL complete RPMI‐1640 medium. The cells (2 × 10^5^ cells/mL) seeded in 25‐cm^2^ cell culture flasks with 5‐mL complete RPMI‐1640 medium. In this way, cells were subcultured from Passage 3 to Passage 24. Serial passaging was performed using a single‐cell lineage; no independent biological replicates were conducted, as the aim was to track within‐lineage mutation dynamics.

### 2.2. Cell Culture Supernatant and Cell Pellet Collection

Cell culture supernatants and cell pellets were collected from each passage to extract RNA. Samples from each passage were tested in technical duplicates (two independent RT‐PCRs per sample). Negative results were confirmed by at least two independent RT‐PCR assays. Cell culture supernatants were centrifuged at 12000 rpm for 10 min to remove any remaining cell debris or particulate matter, then were transferred into the centrifuge tube and stored at −80°C. Cell suspensions were centrifuged at 1000 rpm for 5 min, and the supernatants were discarded. The cell pellets were washed twice with PBS and then stored at −80°C.

### 2.3. Total RNA Isolation and cDNA Synthesis

Total RNA was isolated with the use of a commercial kit (9109, Takara, Japan). In brief, 1 mL of RNAiso Plus was added to the 700‐μL cell culture supernatants or 6 × 10^6^ cell pellets, and then, total RNA was extracted according to the manufacturer’s protocol. The concentration of total RNA was determined using a NanoDrop 2000 spectrophotometer (Thermo Fisher Scientific, USA). cDNA was synthesized from the isolated total RNA (1 μg RNA in 20‐μL reaction systems) using PrimeScript RT reagent Kit with gDNA Eraser (RR047A, Takara, Japan), according to the manufacturer’s protocol.

### 2.4. ENTV‐2 RNA Detection

ENTV‐2 RNA was detected in cell culture supernatants and cell pellets by RT‐PCR. The PCRs were performed using a protocol that amplifies an 814‐bp sequence using primer pairs E814‐F 5′‐TCCACC CTTCCTGGTGCC‐3′ and E814‐R 5′‐CACAAACATGCCCTCGTCCCC‐3’ (Li et al., 2022). The reaction systems were prepared according to the manufacturer’s protocol of 2 × Es Taq MasterMix (Dye) (CW0690M, CoWin Biosciences, Inc., China). The conditions were as follows: denaturation for 2 min at 94°C, and 40 amplification cycles (94°C for 15 s, 56°C for 15 s, 72°C for 45 s) followed by a final extension at 72°C for 5 min. PCR products were detected by electrophoresis on a 1% agarose gel and were photographed by means of an imaging system (ChemiDoc, Bio‐Rad Laboratories, Inc.). A sample was scored as positive when a specific amplicon of the expected size (814 bp) was observed in both technical replicates.

### 2.5. Cloning of ENTV *Env* Fragments and Sequence Analysis

The *env* fragments were amplified using primer pairs env F 5′‐GGGACGAGGGCATGTTTGT GTTTTTC‐3′ and env R 5′‐CCCAGGACTTAACCCTTCAT GGG‐3′ designed by SnapGene Software according to the sequence of ENTV‐2 (LC762617.1). The reaction systems and conditions were similar to those of the above ENTV‐2 RNA detection (94°C for 15 s, 60°C for 15 s, 72°C for 2 min). The PCR products were purified using the TIANgel Purification Kit (GDP219; Tiangen Biotech [Beijing] Co., Ltd., China) and cloned into the pMD 19‐T Vector (6013, Takara, Japan) according to the manufacturer’s protocol. For each selected passage, one positive clone was picked and subjected to Sanger sequencing (Sangon Biotech Co., Ltd., Shanghai, China). All reported mutations were confirmed by bidirectional Sanger sequencing of the single clone from each passage. Nucleotide sequence assembly, sequence alignment, and amino acid sequence prediction were performed using the SeqMan, MegAlign, and EditSeq programs in the DNASTAR software package (DNASTAR Inc., USA). We acknowledge that sequencing of only one clone per passage limits the ability to assess intrapopulation sequence heterogeneity; this limitation is addressed in the Discussion.

### 2.6. Prediction of Secondary Structure of Env Protein

The TMHMM 2.0 online server (https://services.healthtech.dtu.dk/services/TMHMM-2.0) was employed to predict transmembrane helix regions of proteins. The PSIPRED 4.0 online prediction platform (https://bioinf.cs.ucl.ac.uk/psipred) was utilized to predict the secondary structure of the Env protein encoded by the *env* gene, obtaining the composition ratio of conformational states predicted by various algorithms and their sequence localization. The Tencent iDrug online platform (https://drug.ai.tencent.com) was used to convert FASTA sequences of Env protein into PDB format, and AI‐assisted homology modeling algorithms were employed to complete structural predictions.

PyMOL software was applied to perform 3D structural rendering of Env proteins. The Passage 3 Env protein was used as the reference structure for root‐mean‐square deviation (RMSD) comparisons, as Passage 3 represented the earliest passage in this experimental series and the closest available proxy for the original (near‐primary) cell population. Multigeneration protein structure comparisons were conducted based on this reference to obtain the total RMSD values. Nonsynonymous mutation sites were marked using the “create” command, with mutated residues displayed as stick models.

## 3. Results

### 3.1. Detection of ENTV‐2 Across Cell Passages

**FIGURE 1:**
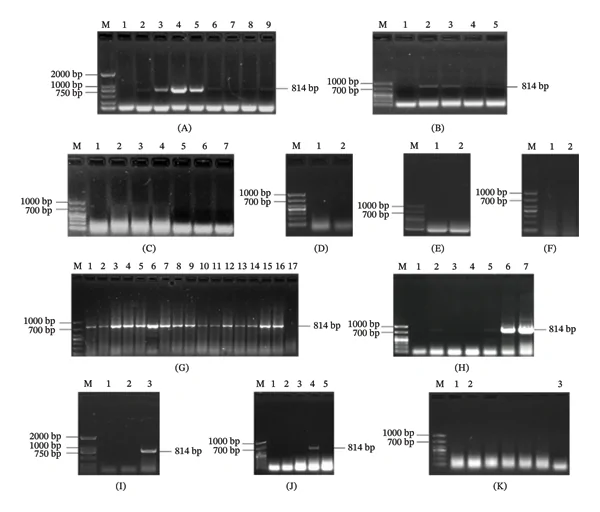
ENTV‐2 PCR detection in supernatants and cell pellets (P3–P24) (A) 1: negative control; 2–9: supernatants from Passages 3 to 10. (B) 1: negative control; 2–3: supernatants from Passage 11; 4–5: supernatants from Passage 12. (C) 1: negative control; 2–4: supernatants from Passage 13; 5–7: supernatants from Passage 14. (D) 1: negative control; 2: supernatants from Passage 15. (E) 1: negative control; 2: supernatants from Passage 16. (F) 1: negative control; 2: supernatants from Passage 17. (G) 1: negative control; 2–17: cell pellets from Passages 3 to 18. (H) 1: negative control; 2: supernatants from Passage 18; 3–4: supernatants from Passage 20; 5: supernatants from Passage 19; 6: cell pellets from Passage 19; 7: cell pellets from Passage 20. (I) 1: negative control; 2: supernatants from Passage 21; 3: cell pellets from Passage 21. (J) 1: negative control; 2: supernatants from Passage 22; 3: supernatants from Passage 23; 4: cell pellets from Passage 22; 5: cell pellets from Passage 23. (K) 1: supernatants from Passage 24; 2: cell pellets from Passage 24; 3: negative control.

Figure 1 shows the detection of ENTV‐2 in supernatants and cell pellets of ENA‐1 cells from Passages 3 to 24. The supernatants from Passages 3–12, 18, and 19 were tested positive for ENTV‐2, while those from Passage 20 onward were negative. In contrast, the cell pellets were positive from Passages 3 to 22, becoming negative at Passages 23 and 24. These results indicate that the ENA‐1 cell line gradually ceased to shed the virus during serial passaging, with viral RNA becoming undetectable by RT‐PCR after Passage 23.

### 3.2. Cloning and Sequencing Analysis of the ENTV‐2 *Env* Gene

PCR products of 1896 bp, amplified from cell pellets at eleven different passages (3, 5, 7, 9, 11, 13, 15, 17, 19, 21, and 22), were cloned into the pMD 19‐T Vector for sequencing. The resulting sequences represented the complete *env* gene for each passage. The full‐length *env* gene mRNA is 1869 nt long and encodes a 622‐amino acid protein.

Sequence alignment analysis using MegAlign software revealed no insertions or deletions in the *env* gene during passaging. However, nucleotide mutations were detected. A total of 28 base mutations, point mutations, were identified when compared to the Passage 3 reference sequence. The distribution of these mutations across passages is summarized in Table 1, with each passage carrying 4 to 8 mutations relative to Passage 3. The predominant mutation type was A ⟶ G transition (18 occurrences, 64.286%), followed by G ⟶ A (5 occurrences, 17.857%) and T ⟶ C (5 occurrences, 17.857%). Among these, 16 base substitutions resulted in changes to the amino acid residues, while the remaining mutations were silent, causing no alteration to the encoded amino acids.

**TABLE 1:** Nucleotide and amino acid mutations in the ENTV‐2 *env* gene during serial passaging.

Genomic position (nt)	Passage											Mutation (base)	Mutation (amino acid)
	3	5	7	9	11	13	15	17	19	21	22		
57	G	G	G	G	G	G	**A**	G	G	G	G	G ⟶ A	**W** ⟶ ^∗^
91	A	A	A	A	A	A	A	**G**	A	A	A	A ⟶ G	M ⟶ V
139	A	A	A	A	A	A	A	**G**	A	A	A	A ⟶ G	R ⟶ G
172	A	A	A	A	**G**	A	A	A	A	A	A	A ⟶ G	N ⟶ D
264	A	**G**	A	A	A	A	A	A	A	A	A	A ⟶ G	/
444	T	T	T	T	T	T	T	T	T	**C**	T	T ⟶ C	/
503	A	**G**	**G**	**G**	**G**	**G**	**G**	**G**	G	**G**	**G**	A ⟶ G	Q ⟶ R
692	G	G	**A**	G	G	G	G	G	G	G	G	G ⟶ A	**W** ⟶ ^∗^
735	A	**G**	A	A	A	A	A	A	A	A	A	A ⟶ G	/
762	A	A	A	**G**	A	A	A	A	A	A	A	A ⟶ G	/
776	T	**C**	T	T	T	T	T	T	T	T	T	T ⟶ C	I ⟶ T
882	G	G	**A**	G	G	G	G	G	G	G	G	G ⟶ A	/
944	A	A	A	A	A	A	A	**G**	A	A	A	A ⟶ G	E ⟶ G
1233	A	A	A	A	A	A	A	A	A	**G**	A	A ⟶ G	/
1324	A	A	A	A	A	**G**	A	A	A	A	A	A ⟶ G	I ⟶ V
1335	A	A	A	A	A	A	**G**	A	A	A	A	A ⟶ G	/
1369	G	G	G	**A**	G	G	G	G	G	G	G	G ⟶ A	G ⟶ R
1391	A	A	A	A	A	A	**G**	A	A	A	A	A ⟶ G	N ⟶ S
1404	G	**A**	**A**	**A**	**A**	**A**	**A**	**A**	**A**	**A**	**A**	G ⟶ A	/
1466	A	A	A	A	A	A	A	A	A	A	G	A ⟶ G	D ⟶ G
1488	A	A	A	A	A	A	**G**	A	A	A	A	A ⟶ G	/
1507	T	T	T	**C**	T	T	T	T	T	T	T	T ⟶ C	W ⟶ R
1557	T	**C**	T	T	T	T	T	T	T	T	T	T ⟶ C	/
1652	A	A	A	**G**	A	A	A	A	A	A	A	A ⟶ G	H ⟶ R
1672	T	**C**	**C**	**C**	**C**	**C**	C	**C**	**C**	**C**	**C**	T ⟶ C	/
1767	A	A	A	A	A	A	A	**G**	A	A	A	A ⟶ G	/
1841	A	A	A	**G**	A	A	A	A	A	A	A	A ⟶ G	E ⟶ G
1859	A	A	A	A	A	A	A	A	A	**G**	A	A ⟶ G	D ⟶ G

*Note:* Bold indicates the nucleotide or amino acid that differs from the reference sequence.

^∗^Stop codon; / indicates synonymous mutation.

Notably, two nonsynonymous mutations led to the formation of premature termination codons: a G ⟶ A mutation at Position 692 in the *env* sequence from Passage 7 generated a stop codon (TAG), and a G ⟶ A mutation at Position 57 in the *env* sequence from Passage 15 created a stop codon (TGA). Additionally, a stable mutation was observed at nucleotide Position 503 of the *env* gene, where an A in Passage 3 was consistently replaced by G from Passage 5 onward, resulting in an amino acid change from glutamine (Q) to arginine (R). In contrast, the G ⟶ A mutation at Position 1404 (from Passage 5 onward) and the T ⟶ C mutation at Position 1672 (from Passage 5 onward) were both synonymous, introducing no change to the amino acid sequence.

### 3.3. Secondary Structure Prediction of Env Protein

**FIGURE 2:**
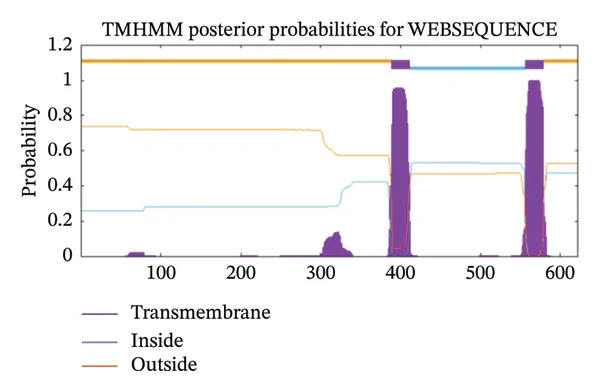
Prediction of transmembrane domains in Env protein.

The transmembrane helix regions of the Passage 3 Env protein were predicted using the TMHMM 2.0 online server, as shown in Figure 2. The Env protein is predicted to be a double‐pass transmembrane protein with a characteristic biphasic transmembrane helix architecture. This structure divides the protein into the following domains: an N‐terminal extracellular domain (1–388), a transmembrane helix (389–411), an intracellular domain (412–555), a second transmembrane helix (556–578), and a C‐terminal extracellular domain (579–622).

As premature translational termination occurred in Passages 7 and 15, protein secondary structure predictions were not performed for them using PSIPRED 4.0. For the remaining passages, nucleotide mutations accumulated during passaging led to variations in the distribution of α‐helix, β‐sheet, and random coil among the Env proteins from different passages (Table 2). However, the impact of these variations on protein stability and function was limited.

**TABLE 2:** Number of amino acids involved in theoretical secondary structure formation in Env protein across different passages.

Passage	α‐helix	β‐sheet	Random coil
3	252	85	285
5	250	79	293
9	257	80	285
11	255	87	280
13	248	84	290
17	253	89	280
19	251	83	288
21	255	82	285
22	251	84	287

Screening of all mutation sites for their locations relative to transmembrane regions revealed that all mutation sites were located in non‐transmembrane regions.

### 3.4. Visualization and Comparative Analysis of Env Protein Structures

**FIGURE 3:**
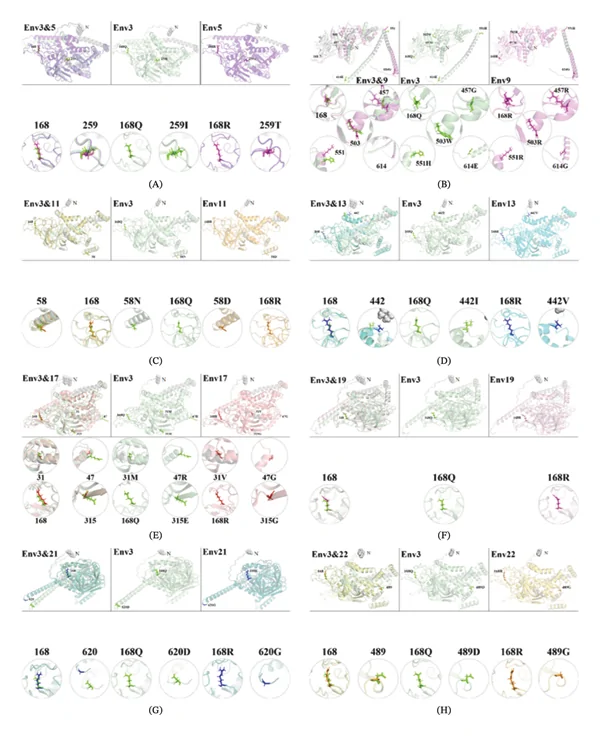
Comparison of the three‐dimensional structures between Env3 and Env proteins of other passages. (A) Env3 vs. Env5. (B) Env3 vs. Env9. (C) Env3 vs. Env11. (D) Env3 vs. Env13. (E) Env3 vs. Env17. (F) Env3 vs. Env19. (G) Env3 vs. Env21. (H) Env3 vs. Env22.

Using PyMOL software, the three‐dimensional structures of Env proteins of other passages were rendered. With the third‐generation Env (Env3) protein as the reference structure, multipassage protein structure alignment was performed using the “align” command, and nonsynonymous mutation sites across passages were labeled. RMSD analysis between the Env3 and Env protein of other passages (Table 3) showed that, except for Env9, the RMSD values of Env proteins from all other passages were below 1 Å, indicating a highly conserved overall three‐dimensional structure and stable core folding pattern across the passages examined. In contrast, the RMSD value for Env9 was 1.306 Å, slightly above 1 Å. An RMSD of 1.306 Å indicates a minor structural deviation; whether this difference is biologically meaningful requires experimental validation. This deviation may reflect local conformational changes without disrupting the global fold (Figure 3). It can be observed that the increased side‐chain volume of the arginine (R) at Position 457 in Env9 may introduce steric hindrance, thereby potentially interfering with the interaction between Env and other proteins.

**TABLE 3:** RMSD values of Env proteins (various passages vs. P3).

Passage	RMSD value (Å)
5	0.842
9	1.306
11	0.544
13	0.688
17	0.896
19	0.874
21	0.939
22	0.869

## 4. Discussion

Currently, an in vitro culture system for ENTV‐2 has not yet been fully established, and stable, suitable cellular models remain lacking. Existing studies indicate that transfection of an infectious clone into HEK 293T cells can successfully generate mature viral particles [8]. Additionally, some researchers have established monoclonal cell lines from tumor tissue; however, the virus could no longer be detected after 17 passages, suggesting that the mechanism sustaining viral persistence in vitro remains unclear [7]. Whether the *env* gene undergoes dynamic evolution to adapt to the cell culture environment during serial passaging, and how such changes might affect viral infectivity and oncogenicity, remains to be investigated.

This study conducted successive passage culture of ENA‐1 cells, systematically monitoring the presence of ENTV‐2 virus in both supernatants and cell pellets. The results showed that ENTV‐2 RNA became progressively undetectable during passaging. After the 13th passage, particularly, the detection of ENTV‐2 in supernatants became inconsistent, and from the 23rd passage onward, both cell pellets and supernatants tested negative for the virus. This phenomenon is highly consistent with observations for jaagsiekte sheep retrovirus (JSRV) and enzootic nasal tumor virus 1 of sheep (ENTV‐1) during in vitro culture [9, 10]. ENTV‐2, along with JSRV and ENTV‐1, belongs to the genus *Betaretrovirus* affecting sheep and goats, sharing a high degree of genomic homology, which suggests common characteristics in their inability to sustain infection in non‐natural host cells in vitro.

Walsh et al. found that while establishing ENTV‐1‐infected cell lines, the majority of single‐cell clones could no longer be detected for proviral DNA by the 5th passage [10]. Notably, even the clones selected for long‐term culture eventually all became negative. This indicates that in the in vitro passage environment, virus‐free cell clones gained a significant growth advantage [10]. Similarly, Palmarini et al. discovered that high‐level replication of JSRV in infected sheep cell lines strictly depended on the specific differentiated state of the host cells. Loss of this state during cell passaging led to proviral silencing or loss [9]. These studies collectively suggest that for JSRV and ENTV, the selective pressures of in vitro culture favor the proliferation of cell clones that either lack the virus or harbor silenced proviruses, allowing them to eventually dominate the cell population. This mechanism may explain the loss of ENTV‐2 observed in our study during successive passaging and is consistent with previous observations in related betaretroviruses. Furthermore, research by Palmarini et al. also found that viral release into the supernatants remained consistently very low, requiring highly sensitive assays for detection. The underlying reason is that the transcriptional activity of the JSRV LTR promoter is highly cell‐type‐specific. In nonoptimal in vitro cultured cells, its transcriptional efficiency is extremely low. This results in inefficient viral transcription, translation, and ultimately assembly and release, even when the proviral DNA is integrated into the host genome [9].

ENTV‐2 is a *Betaretrovirus* with a genome structure composed of four major overlapping gene regions in the order: 5′‐*gag-pro-pol-env*‐3’. The *env* gene encodes the viral Env protein. The precursor Env protein undergoes a two‐step proteolytic cleavage process: First, the N‐terminal signal peptide is removed to direct the protein to the endoplasmic reticulum; subsequently, it is cleaved at an internal RPKR motif to generate the SU and TM subunits [10]. The SU subunit is responsible for binding to the host cell receptor Hyal2, mediating viral entry and activating pro‐proliferative signaling pathways [11]. The TM subunit executes membrane fusion and signal transduction functions, with the YXXM motif within its cytoplasmic tail domain being a critical functional site for oncogenic activity [2, 12]. Therefore, the Env protein plays a crucial role in viral infection of host cells, and its variation may influence viral infectivity and host specificity.

In this study, the *env* gene from cell Passages 3 to 24 was cloned and sequenced. A total of 28 nucleotide mutations were identified in the *env* gene during passaging, including three stable mutations; no nucleotide deletions or insertions occurred. Based on the known structure of the ENTV‐2 *env* gene, analysis of the mutation sites revealed that the nucleotide mutations at Position 57 (Passage 15) and Position 692 (Passage 7) introduced premature termination codons, potentially leading to truncation of the SU subunit, which may affect its binding ability based on structural prediction. Furthermore, the A ⟶ G mutation at Position 503 (Q168R) was fixed across all passages. This site is located in the SU subunit, where a polar glutamine is replaced by a positively charged arginine, which may alter protein surface properties and receptor interactions.

The mutations at Position 1404 (G ⟶ A) and Position 1672 (T ⟶ C) were synonymous mutations located in the TM subunit region, resulting in no amino acid change. However, other nonsynonymous mutations within functional TM domains, such as the T ⟶ C mutation at Position 1507 (W503R), may affect membrane fusion or signal transduction by altering local conformation or charge distribution. Notably, all mutations were located outside transmembrane regions. The sequence changes observed in non‐transmembrane regions are consistent with a possible adjustment of extracellular or intracellular domain structures, although this interpretation is speculative.

Transmembrane prediction for the Env protein indicated a dual‐transmembrane structure in the third‐generation protein. Minor variations in the distribution of α‐helices, β‐sheets, and random coils were observed across passages. Except for the 7th and 15th passages, where nonsense mutations led to protein truncation, structural variations in other passages were minimal. Structural comparison using PyMOL showed that the RMSD values for most Env proteins relative to the third‐generation reference structure were below 1 Å, indicating high conservation of the core structure. The 9th‐passage Env protein exhibited a slightly higher RMSD value (1.306 Å) due to the W503R mutation. Located in the SU subunit, this mutation substitutes hydrophobic tryptophan with positively charged arginine, potentially disrupting local hydrophobic interactions and causing a transition from α‐helix to random coil, thereby affecting SU‐receptor binding. Although the G457R mutation did not induce secondary structural changes, the increased side‐chain volume of arginine may introduce steric hindrance, potentially impacting protein function.

Interpretation of the mutation sites summarized in Table 4 should be considered speculative. Based on computational predictions, the Q168R mutation, which was consistently identified in the sequenced clone from each passage from Passage 5 onward, did not alter the predicted secondary structure; however, the substitution of glutamine by arginine may theoretically affect protein surface charge. The W19^∗^ (Passage 15) and W231^∗^ (Passage 7) mutations are predicted to result in premature truncation of the protein, potentially affecting the extracellular domain. Transient mutations such as W503R (Passage 9) and G457R (Passage 9) appeared only in Passage 9 and were not retained in subsequent passages. Other mutations, including charge‐related substitutions (E614G, E315G, D620G, D489G, N58D, R47G) and hydrophobic substitutions (I259T, I442V, M31V), did not significantly alter the predicted secondary structure. However, these interpretations are based solely on structural predictions, and their biological or functional relevance requires experimental validation.

**TABLE 4:** Analysis of theoretical secondary structures and physicochemical properties at amino acid mutation sites.

Amino acid positions	Passage											Mutation	Alteration in secondary structure	Alteration in physicochemical properties	Located in TRE
	3	5	7	9	11	13	15	17	19	21	22				
19	W	W	W	W	W	W	^∗^	W	W	W	W	W ⟶ ^∗^	/	/	No
31	M	M	M	M	M	M	/	**V**	M	M	M	M ⟶ V	H ⟶ H	Hydrophobic ⟶ Hydrophobic	No
47	R	R	R	R	R	R	/	**G**	R	R	R	R ⟶ G	H ⟶ H	Small nonpolar ⟶ Polar	No
58	N	N	N	N	**D**	N	/	N	N	N	N	N ⟶ D	H ⟶ H	Polar ⟶ Polar	No
168	Q	**R**	**R**	**R**	**R**	**R**	/	**R**	**R**	**R**	**R**	Q ⟶ R	C ⟶ C	Polar ⟶ Polar	No
231	W	W	^∗^	W	W	W	/	W	W	W	W	W ⟶ ^∗^	/	/	No
259	I	**T**	/	I	I	I	/	I	I	I	I	I ⟶ T	C ⟶ C	Hydrophobic ⟶ Small nonpolar	No
315	E	E	/	E	E	E	/	**G**	E	E	E	E ⟶ G	E ⟶ E	Polar ⟶ Small nonpolar	No
442	I	I	/	I	I	**V**	/	I	I	I	I	I ⟶ V	H ⟶ H	Hydrophobic ⟶ Hydrophobic	No
457	G	G	/	**R**	G	G	/	G	G	G	G	G ⟶ R	H ⟶ H	Small nonpolar ⟶ Polar	No
489	D	D	/	D	D	D	/	D	D	D	**G**	D ⟶ G	C ⟶ C	Polar ⟶ Small nonpolar	No
503	W	W	/	**R**	W	W	/	W	W	W	W	W ⟶ R	H ⟶ C	Aromatics plus cysteine ⟶ Polar	No
551	H	H	/	**R**	H	H	/	H	H	H	H	H ⟶ R	H ⟶ H	Polar ⟶ Polar	No
614	E	E	/	**G**	E	E	/	E	E	E	E	E ⟶ G	C ⟶ C	Polar ⟶ Small nonpolar	No
620	D	D	/	D	D	D	/	D	D	**G**	D	D ⟶ G	C ⟶ C	Polar ⟶ Small nonpolar	No

*Note:* / indicates premature termination of protein translation, and thus, no comparison was made; H, α‐helix; E, β‐sheet; C, random coil; TRE, transmembrane region.

^∗^A nonsense mutation resulting in a premature stop codon, which leads to early termination of protein translation.

Study limitations: Several limitations of this study should be acknowledged. First, the passaging was performed using a single‐cell lineage without independent biological replicates. Although biological replicates would have strengthened the generalizability of our observations, our design was intended to track within‐lineage sequence dynamics. The lack of biological replicates is acknowledged as a limitation. Second, viral detection was based on qualitative endpoint RT‐PCR rather than RT‐qPCR; therefore, we report viral RNA detection status rather than absolute viral load. Third, only one clone per passage was sequenced due to the exploratory nature of the study. The mutation data represent single‐clone sequences and require confirmation in additional clones. Fourth, and most importantly, no functional experiments (e.g., infectivity, receptor binding, Env protein expression, membrane fusion, or oncogenicity assays) were performed. Consequently, all evolutionary and functional interpretations presented here are speculative and hypothesis‐generating, and they require experimental validation in future studies. Fifth, because only one cloned PCR product was sequenced per passage, the possibility of PCR‐induced sequence artifacts cannot be completely excluded. PCR amplifications were performed using standard Taq DNA polymerase; all reported mutations were confirmed by bidirectional Sanger sequencing of the single clone, but sequencing of additional clones would be required to rule out PCR‐associated errors. We emphasize that our findings should be interpreted as descriptive sequence observations rather than confirmatory evidence of functional adaptation.

## Author Contributions

Study design: Mingxiao Li and Dawei Yao.

Data collection: Mingxiao Li, Ziyan Lin, Lingxu Li, Zhen Wang, Bingyan Wei, and Guangjun Chang.

Statistical analysis: Mingxiao Li, Wenqing Guo, and Guangjun Chang.

Data interpretation: Mingxiao Li, Wenqing Guo, Guangjun Chang, and Dawei Yao.

Manuscript preparation: Mingxiao Li, Wenqing Guo, and Dawei Yao.

Literature search: Mingxiao Li, Ziyan Lin, and Lingxu Li.

Laboratory studies: Mingxiao Li, Zhen Wang, and Bingyan Wei.

## Funding

No funding was received for this manuscript.

## Conflicts of Interest

The authors declare no conflicts of interest.

## Data Availability

The datasets used and/or analyzed during the current study are available from the corresponding author upon reasonable request.
